# Japanese Version of the Mobile App Rating Scale (MARS): Development and Validation

**DOI:** 10.2196/33725

**Published:** 2022-04-14

**Authors:** Kazumichi Yamamoto, Masami Ito, Masatsugu Sakata, Shiho Koizumi, Mizuho Hashisako, Masaaki Sato, Stoyan R Stoyanov, Toshi A Furukawa

**Affiliations:** 1 Departments of Health Promotion and Human Behavior Kyoto University Graduate School of Medicine School of Public Health Kyoto Japan; 2 Research Unit Institute for Airway Disease Takarazuka Japan; 3 Department of Health Informatics Kyoto University Graduate School of Medicine School of Public Health Kyoto Japan; 4 Department of Sociology Rikkyo University Tokyo Japan; 5 Organ Transplantation Center The University of Tokyo Hospital Tokyo Japan; 6 Institute of Health & Biomedical Innovation, School of Psychology and Counselling Queensland University of Technology Brisbane Australia

**Keywords:** mobile health apps, MHAs, mHealth, mobile application, mobile application rating scale, MARS, scale development, mental health, mobile health applications

## Abstract

**Background:**

The number of mobile health (mHealth) apps continues to rise each year. Widespread use of the Mobile App Rating Scale (MARS) has allowed objective and multidimensional evaluation of the quality of these apps. However, no Japanese version of MARS has been made available to date.

**Objective:**

The purposes of this study were (1) to develop a Japanese version of MARS and (2) to assess the translated version’s reliability and validity in evaluating mHealth apps.

**Methods:**

To develop the Japanese version of MARS, cross-cultural adaptation was used using a universalist approach. A total of 50 mental health apps were evaluated by 2 independent raters. Internal consistency and interrater reliability were then calculated. Convergent and divergent validity were assessed using multitrait scaling analysis and concurrent validity.

**Results:**

After cross-cultural adaptation, all 23 items from the original MARS were included in the Japanese version. Following translation, back-translation, and review by the author of the original MARS, a Japanese version of MARS was finalized. Internal consistency was acceptable by all subscales of objective and subjective quality (Cronbach α=.78-.89). Interrater reliability was deemed acceptable, with the intraclass correlation coefficient (ICC) ranging from 0.61 to 0.79 for all subscales, except for “functionality,” which had an ICC of 0.40. Convergent/divergent validity and concurrent validity were also considered acceptable. The rate of missing responses was high in several items in the “information” subscale.

**Conclusions:**

A Japanese version of MARS was developed and shown to be reliable and valid to a degree that was comparable to the original MARS. This Japanese version of MARS can be used as a standard to evaluate the quality and credibility of mHealth apps.

## Introduction

Smartphones are now an indispensable part of our lives. According to a 2021 global survey, more than 7.5 billion smartphones are in use around the world, and that number is only expected to increase [1]. With their growing popularity, they have come to have widespread applications in health care in many countries, including Japan [2]. The number of mobile health (mHealth) apps also continues to rise, especially since the beginning of the COVID-19 pandemic in early 2020 [3].

Although an increasing quantity of research showcases the efficacy of mHealth apps in many conditions, such as diabetes mellitus, asthma, and mental health [4-6], the overall evidence on their usefulness remains inconsistent [7]. This may reflect the lack of systematic research on the quality and efficacy of mHealth apps. [8,9].

To date, several medical societies [10,11] and researchers [12] have proposed ways to evaluate mHealth apps. Of these, the Mobile App Rating Scale (MARS) [13] is one of the most comprehensive, simple, and reliable. MARS is a 23-item scale, comprising 4 objective subscales and 1 subjective subscale (described in detail below). Validity and reliability are well supported for this scale [14], and an increasing number of studies use it to evaluate a wide range of mHealth apps [15-20].

The original version of MARS was developed in English, and several validated translations are available, including Italian, Spanish, German, French, and Arabic [21-25]. However, it has yet to be translated into any East Asian language, including Japanese, despite the recent increase in popularity of mHealth apps in this region. The development of standardized evaluation criteria shared among diverse cultures can contribute to the global public benefit of mHealth apps. Nevertheless, to date, no Japanese app evaluation scale exists.

The translation of scales involves not only a direct translation, but also adaptation of the questions to account for cultural differences, followed by appropriate measurements of reliability and validity [26]. The aims of this study were (1) to develop a Japanese version of MARS based on cross-cultural adaptation and (2) to assess the reliability and validity of this Japanese version by evaluating mHealth apps in the Japanese language.

## Methods

### Study Design

This study was conducted in two steps, following the methodology of previous translation and validation studies of the English MARS in other languages [21-25]: (1) cross-cultural adaptation with translation and back-translation and (2) a statistical evaluation of the reliability and validity of the translated scale.

### MARS

The original MARS was developed by Stoyanov and colleagues [13] to establish a multidimensional measure able to classify and evaluate the objective and subjective quality of mHealth apps. The main part of this original version of MARS consisted of 23 items. The objective evaluation of mHealth app quality included 4 subscales: engagement (items 1-5), functionality (items 6-9), aesthetics (items 10-12), and information (items 13-19). The subjective quality subscale consisted of 4 items (items 20-23). Each MARS item is rated on a 5-point Likert scale (from 1 to 5: inadequate, poor, acceptable, good, and excellent), except for items 14 to 17 and item 19, which also have a “not applicable” option, for cases in which the item is not applicable to the evaluation. A mean score for each of the 4 objective subscales and an overall mean score of these 4 subscales are used. To determine subjective quality, individual scores for each item and a mean score for this subscale are rated separately. In addition to these 23 MARS items, sections to rate the classification, description, and perceived impact of the mHealth app can be adjusted according to the aims of the researcher. Both the original MARS [13] and several translated versions [21-25] have been assessed as providing high to satisfactory reliability and validity.

### Cross-Cultural Adaptation and Translation Process

For the adaptation process, we were especially concerned about the cultural and linguistic differences between English and Japanese. Most of the existing translations of MARS are in European languages, which share some degree of cultural and linguistic similarities with English, but not with Japanese [27-29]. Therefore, we decided to adopt the “universalist” approach described by Herdman et al [30]. In this approach, 6 domains are considered for cross-cultural adaptation: item, conceptual, semantic, operational, measurement, and functional equivalence. Following these guidelines, each item and subcategory was assessed by a panel of 4 of the authors, comprising several specialties: a psychologist with a background in epidemiology (M Sakata), a registered nurse with a background in epidemiology (MI), a medical doctor and information technology developer (KY), and a sociologist specializing in questionnaire development (MH). All members are multilingual in Japanese, English, and other languages.

With the agreement of the panel, 3 translations were independently prepared by 3 panel members (M Sakata, MI, and KY). Following review and discussion of the differences between the 3 translations, a first draft of the Japanese translation was developed. This draft version was then back-translated into English, without referencing the original MARS scale or the original article, by a professional Japanese medical translator with a background in clinical epidemiology (SK). The back-translated version was proofread by a native English translator with a background in clinical pharmacy and clinical pharmacology. It was then reviewed and compared to the original by the developer of MARS (SS) and adjusted based on his feedback (Multimedia Appendix 1).

### App Selection and Assessment

To better compare the results of this study with those of the original MARS, we tried to follow the original strategy for app selection and assessment. A systematic search was conducted on the Google Play Store and Apple App Store for mental health apps. The inclusion criteria were as follows: (1) the app was in Japanese, (2) the app was free, (3) the app was designed for adults, and (4) the app was developed by an entity based in Japan. The exclusion criteria were as follows: (1) the app required the registration of personal information, (2) the app was unrelated to health, and (3) the app was developed for ongoing research by another academic entity. Because logic operators (AND, OR, and NOT) are not allowed in the Google Play Store or Apple App Store, the following keywords were used individually: “mindfulness,” “depression,” “wellbeing,” “well-being,” “mental health,” “anger,” “CBT,” “stress,” “distress,” and “anxiety.”

The sample size was calculated based on previous research [12,13,21]. A total of 41 apps were required to demonstrate interrater reliability within 0.15 of a sample observation of 0.80, with 87% assurance (based on 10,000 simulation runs) [31]. Ten apps were evaluated for the training stage, and to account for possible ineligible samples, a sample size of 60 apps was considered necessary for this study. If more than 60 apps were eligible after the systematic search, 60 apps were randomly selected using a random sequence. If an app turned out to be ineligible for evaluation, it was eliminated and another app randomly selected from among the remaining eligible apps.

After watching a training video provided by the author of the original MARS (SS), 10 apps were rated independently by 3 raters (MI, KY, M Sakata) as a training exercise. Then, disagreements were discussed until a consensus was reached to ensure consistent interpretation of all MARS terminology and item logic. Two raters independently assessed the remaining 50 apps in the final analysis.

### Statistical Analysis

#### Descriptive Statistics

The distribution of summary scores (for the total and subscale scores for objective quality) was visually inspected and evaluated for a normal distribution using skewness and the Shapiro-Wilk test. Skewness was judged significant if the estimate was more than plus or minus 1.0. Normally distributed data were expressed as the mean (SD). Floor or ceiling effects were judged to be present if more than 15% of the apps were rated as the lowest or highest scores, respectively.

#### Reliability

The internal consistency of the total and subscale scores for objective quality was assessed using Cronbach α. Internal consistency was deemed acceptable at α>.6 [32]. The interrater reliability was assessed using the intraclass correlation coefficient (ICC) using 2-way mixed effects and an averaged-measurements model with absolute agreement [13,21,22]. ICC was judged acceptable at >0.5 [33].

#### Validity

For construct validity, item-subscale correlations were investigated using multitrait scaling analysis [34]. The convergent validity was deemed satisfactory if the item achieved at least a correlation of 0.4 with its item-own subscale. For discriminant validity, the correlation coefficients of each item with an item-own subscale were compared with those with other subscales. The discriminant validity was considered satisfactory if more than 80% of correlation coefficients in the item-own subscale were higher than those with other subscales [22]. We expressed these estimates as the success rate—the number of items that fulfilled the above-mentioned conditions, divided by the total number of items within the subscale. This success rate was only calculated for the 4 objective quality subscales, because subjective quality is rated independently from objective quality in MARS.

To determine concurrent validity, the lack of an external “gold standard” rating scale led us to compare the correlation between the mean scores from 4 subscales of objective quality against the star rating and subjective quality total mean score using the Pearson *r* coefficient with 95% CI. The correlation between the mean total score of objective quality and mean star ratings in the app stores was also determined as in the original MARS [13].

#### Statistical Software

R (version 4.0.5; R Foundation for Statistical Computing) was used for all analyses.

## Results

### Cross-Cultural Adaptation and Translation Process

The 4 specialists held a joint discussion to conduct a conceptual analysis of the Japanese translation. All subscales and items were evaluated for conceptual equivalence between English and Japanese. The panel agreed to include all items in all of the subscales in the translation.

No major discrepancies were found among the 3 independently developed translations. All differences in expression were resolved through discussion. However, we encountered issues when translating several words that had no Japanese equivalent. For example, for the word “engagement,” it seemed that no Japanese word could express this concept. In such cases, we translated the word into terms as close as possible to the original concept together with the phonetic rendition in *katakana*, a Japanese syllabary used to express foreign words based on their pronunciation.

After creating the initial Japanese version of MARS, a back-translation was sent to the author of the original MARS without modifications. In general, the back-translated version was deemed equivalent to the original MARS. Several comments were provided to clarify word meanings. All comments from the original MARS author were reviewed and integrated, where relevant, by the 4 researchers who developed the Japanese MARS together with the translator who provided the back-translation (SK). Because the back-translation was considered appropriate and no major changes were made, no second back-translation was created after discussion with the author of the original MARS (SS).

### App Selection and Test Phase

**Figure 1:**
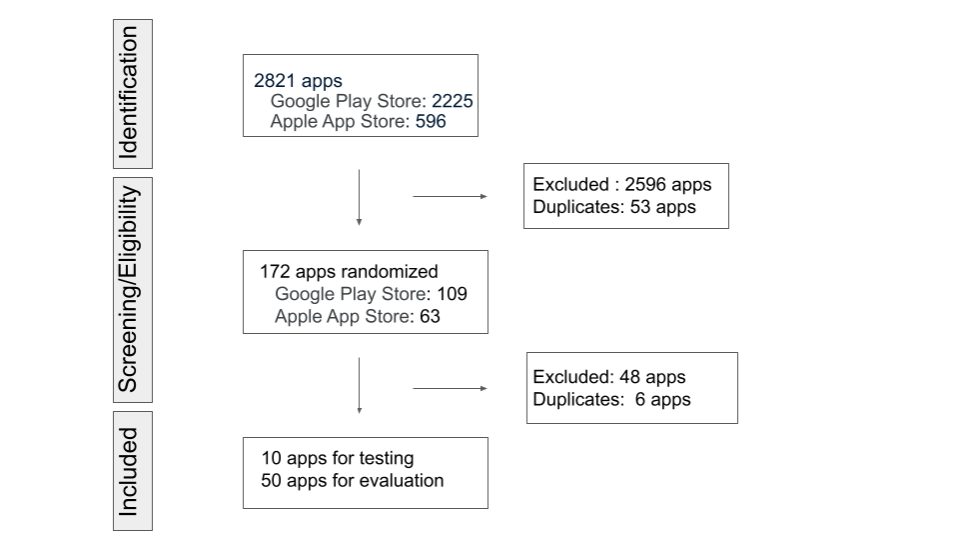
Flow diagram showing the process of identifying apps for pilot use of the Mobile App Rating Scale (MARS).

A search of the Apple App Store and Google Play Store was conducted on June 4 and June 11, 2021. A total of 2821 apps (Apple App Store: n=596; Google Play Store: n=2225) were retrieved. All the apps were screened for adherence to the inclusion and exclusion criteria based on the information page for the app. Of 225 candidate apps, 53 were duplicates, and the remaining 172 apps were the final candidates for random sampling. A computer-generated random sequence was assigned, and the first 60 apps were selected for testing and evaluation (Figure 1). Fifty-four apps were excluded from the list during this rating phase based on the inclusion and exclusion criteria.

### Reliability and Validity Analysis

Among the 50 apps analyzed, 36 (72%) were from the Google Play Store, and 14 (28%) were from the Apple App Store. A response of “not applicable (N/A)” was allowed for items 14 to 17 and 19 when there were no concrete goals (item 14), no information (items 15-17), or no search results in Google Scholar (item 19). More than 50% of the values for these items were therefore missing (73%, 60%, 61%, 58%, and 91% for items 14 to 17 and 19, respectively). It was decided to treat these values as missing in most of the analyses, except for the item-subscale correlation analysis, where a value of zero was assigned as “not applicable.”

Table 1 shows the descriptive analysis results. No skewness was apparent in subscale score distributions. The Shapiro-Wilk test revealed a lack of fit to a normal distribution in several subscales. However, after visual inspection of the distributions, the mean (SD) was finally determined for all subscales. No ceiling or floor effects were detected.

Table 2 shows the results of the reliability analysis. Cronbach α was deemed acceptable in all objective and subjective quality subscales, with a range of α=.78 to .89. ICC results were considered acceptable for all subscales of objective quality and subjective quality, falling within the range of 0.61 to 0.79, except for the “functionality” subscale, which had an ICC of 0.40 (95% CI 0.20-0.54).

**Figure 2:**
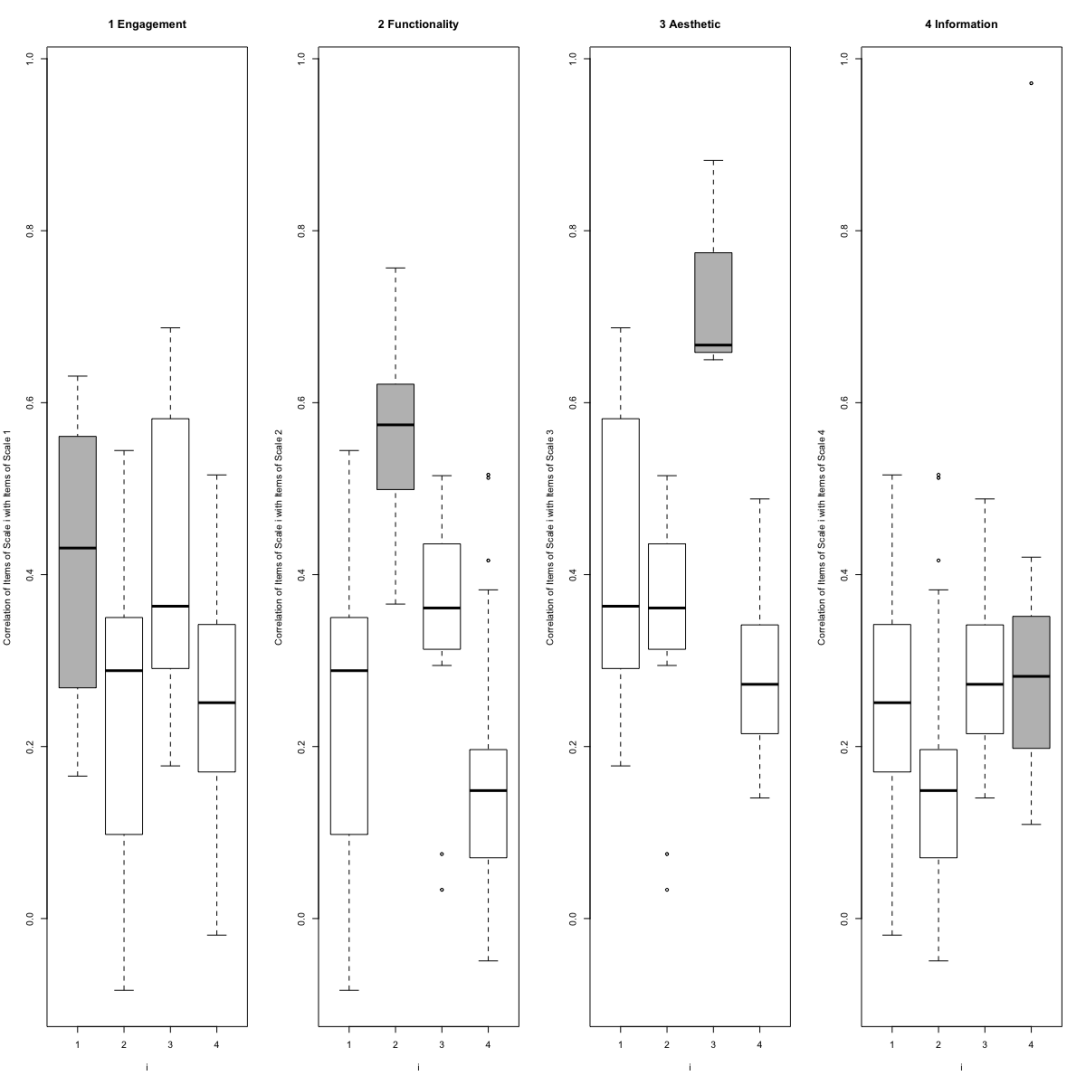
Box plots of subscale correlations with item-own and other subscales. The mean correlation of each subscale is higher than the correlation with other subscales.

As shown in Table 3, the results of convergent and divergent validity were analyzed using multitrait scaling analysis. Item 19 was eliminated from the analysis because more than 90% of responses were “not applicable.” As for convergent validity, most items were deemed acceptable with a correlation of >0.4, and the success rate was satisfactory, except for the subscale “information” (50%). For divergent validity, most items were satisfactory, with more than an 80% success rate, except for the subscale “information” (67%). Figure 2 shows a visual image of item-subscale relationships in subscales of objective quality.

Table 4 shows the concurrent validity based on the Pearson correlation coefficient between the total score (ie, the combined scores for objective and subjective quality) vs the MARS star rating (item 23) and the star rating on the app stores (ie, Google Play Store and Apple App Store). A statistically significant correlation was found between the total score and the MARS star rating at >0.8 with a relatively narrow 95% CI. However, this correlation was not observed in the correlation between the total score and the app store star rating (0.17-0.3), which had a wider 95% CI.

**Table 1:** Descriptive statistics.

Scale		Skewness	Shapiro-Wilk (*P*)	Ceiling effect (%)	Floor effect (%)	Mean (SD)
**Objective quality**						
	Engagement	0.25	0.98 (.16)	1	2	2.64 (0.74)
	Functionality	–0.96	0.93 (<.001)	2	2	3.67 (0.82)
	Aesthetics	0.21	0.96 (.002)	4	3	3.13 (0.83)
	Information	–0.29	0.97 (.06)	1	2	2.98 (0.69)
	Total Score	–0.16	0.99 (.32)	1	1	2.90 (0.63)
Subjective quality		0.53	0.93 (<.001)	1	14	2.20 (0.94)

**Table 2:** Internal consistency and interrater reliability.

Scale		Cronbach α		Intraclass correlation coefficient (95% CI)
**Objective quality**				
	Engagement	.78	0.69 (0.57-0.77)	
	Functionality	.83	0.40 (0.20-0.54)	
	Aesthetics	.89	0.61 (0.4-0.72)	
	Information	.82	0.79 (0.23-0.75)	
	Total Score	.81	0.70 (0.65-0.74)	
Subjective quality		.88		0.75 (0.67–0.81)

**Table 3:** Construct validity measured with multitrait scaling analysis.

Subscale and item		Corrected item-subscale correlation	Success rate^a^	
			Convergent validity	Divergent validity
**Engagement**			4/5	4/5
	Item 1	0.35		
	Item 2	0.61		
	Item 3	0.65		
	Item 4	0.53		
	Item 5	0.62		
**Functionality**			4/4	4/4
	Item 6	0.59		
	Item 7	0.55		
	Item 8	0.81		
	Item 9	0.73		
**Aesthetics**			3/3	3/3
	Item 10	0.68		
	Item 11	0.84		
	Item 12	0.83		
**Information**			3/6	4/6
	Item 13	0.24		
	Item 14	0.33		
	Item 15	0.74		
	Item 16	0.75		
	Item 17	0.39		
	Item 18	0.49		
	Item 19^b^	—^c^	—	—
**Subjective quality^d^**				
	Item 20	0.83	—	—
	Item 21	0.84	—	—
	Item 22	0.55	—	—
	Item 23	0.78	—	—

^a^Success rate was defined as the rate of prespecified acceptable items among all items in each subscale.

^b^Item 19 was eliminated from the analysis because of missing values.

^c^Not applicable.

^d^Success rate was not calculated for subjective quality.

**Table 4:** Concurrent validity of total score measured with the Pearson correlation coefficient.

Scale	Pearson *r*	95% CI	*P* value
Total score vs subjective quality	0.85	0.79-0.90	<.001
Total score vs star rating (item 23)	0.84	0.77-0.89	<.001
Total score vs star rating (app stores)	0.24	0.03-0.42	.02

## Discussion

### Main Study

To our knowledge, this is the first time a cross-cultural approach has been used in the development and validation of a Japanese version of the MARS. This study also includes the involvement of one of the authors of the original MARS. It provides a statistical evaluation of the reliability and validity of the Japanese version in assessing 50 apps in Japanese.

We adopted the universalist approach [30] following practices from previous studies on translations and cross-cultural validation of MARS in other languages. There is controversy about whether it is preferable to adopt a universalist or country-specific approach to patient-reported outcome measures. However, the consortium that qualifies patient-reported outcomes for use in clinical trials in the United States prefers a universalist approach to minimize the variability of language-related logistical complexity [35]. The universalist approach has substantial advantages in achieving conceptual equivalence in cross-cultural translation. Following this approach, we formed a panel with members from a wide variety of disciplines, not only limited to medicine or psychology, but also including an information technology developer, a professional translator with a background in epidemiology, and a sociologist specializing in the development of questionnaires. After discussion within the panel to account for linguistic differences between Japanese and the original English version, we finally decided to include all items with minor modifications for the Japanese version, based on conceptual, semantic, operational, measurement, and functional equivalence. For a cultural adaptation, we believe that it is most practical and helpful to involve specialists from a broad range of backgrounds.

During app selection, 172 apps were found eligible for evaluation, of which 60 were selected. Surprisingly, more than 90% of these apps lacked any scientific evidence supporting them; we found neither research nor supporting articles on their efficacy. Takashina et al [36] evaluated 47 apps for depression that had been developed in Japanese and concluded that very few apps were evidence-based and secure. This situation is quite problematic, because inaccurate or misleading apps could potentially impair the health of users or lead to incorrect decision-making [22]. For this reason, the current study offers a step in the right direction by translating a well-established quality evaluation scale into Japanese.

The analysis of reliability and validity suggests that our results are comparable with the original MARS and other translated versions [13,21-24]. The internal consistency and Cronbach α of all subscales and total scores were satisfactory according to the internationally established quality criteria [37]. This high internal consistency was also observed in the original MARS study and in previous translation studies. Conversely, our study showed slight variability in interrater reliability, with an ICC in the range of 0.40 to 0.79. This finding was also observed in the original MARS, which had a range of 0.50 to 0.83. We evaluated 10 apps after watching a training video provided by the author of the original MARS; 2 raters then discussed the evaluation. Disagreements were discussed until a consensus was reached. We still found low ICC for the “functionality” subscale, however. In that sense, we consider that a test phase and use of a training video are particularly important in assuring mHealth apps are rated correctly.

As for construct validity, we used a multitrait scaling analysis with item-subscale correlation rather than a factor analysis, because this method has been successfully applied in all previous studies. Our results were satisfactory in terms of convergent/divergent validity and fulfilled the prespecified success level, except for the “information” subscale, in which “not applicable” was the choice for most items. This was assigned a value of zero instead of being reported as a missing value. As in the original MARS study, the Japanese version also accepts “not applicable” as a response to items 14 to 17 and 19. During the evaluation, we frequently encountered apps where no clear goal was stated and no information on the source or detailed explanations were provided. In these cases, “not applicable” was chosen rather than one of the choices of the Likert scale. MARS itself takes such situations into account and uses the mean of the subscale total score. However, this is a problem for validation because the proportion of “not applicable” answers exceeded 50% for items 14 to 17 and 19. As a way of resolving this, we assigned zero as the numerical score for “not applicable” rather than treating these as missing values in items 14 to 17, thus allowing a comparison of the proximity of the scores between the raters. Item 19 was eliminated from the analysis, as it was in other MARS translation studies, because mHealth apps mostly lack evidence-based evaluation research, which the item aims to measure. We believe this should be clarified in a future updated version once a better practice for mHealth evaluation is widely implemented.

When measuring concurrent validity, the MARS objective quality total score was significantly and closely correlated with the subjective quality total score and star rating (item 23), with Pearson *r*>0.8. However, it was fairly well correlated with the star rating on the app stores. This finding has also been seen in previous studies [13,22]. As Stoyanov et al [13] reported, it is possible that the MARS subjective quality rating may be influenced by the completion of the MARS objective quality rating, and the results should be evaluated with caution. However, the lack of reliability of the star ratings on app stores has also been reported [38], and in this sense, MARS subjective score or star ratings could be a more reliable indicator of the ratings of mHealth apps.

### Limitations

This study has several limitations. First, this was a validation study that tested only mental health apps. This was to maintain comparability with the original MARS study, which also studied only mental health mHealth apps. However, other translated MARS studies have used apps on other topics, such as physical activity [21] and primary prevention [22]. Accumulating evidence in recent publications shows that MARS is being used to evaluate mHealth apps in a wide variety of areas [15-20]. Thus, availability of a Japanese MARS will facilitate further research on app validation. Secondly, as mentioned above, the “information” subscale could not be adequately validated in this study. Neither the original MARS study nor other translation studies have had missing values, except for item 19, which estimates the degree of the evidence base of an app. However, several items do allow the “not applicable” choice and there are no clearly defined guidelines on the appropriate use of this rating option in the original MARS version. For this reason, it may be prudent to specify standards on choosing this option in future updated versions.

### Future Research

Based on the results of the present study, we would like to propose several topics for future research. First, as made apparent in this study, validation requires further research. In almost all previous studies, item 19 (ie, the evidence base) was excluded from the analysis because of missing data. MARS was created to take missing values into account and uses mean scores instead of sum scores. This, however, makes it complicated to estimate the validity of individual items; more research needs to be performed to validate the scale. Second, a more detailed validation of the Japanese version of MARS is also required, especially regarding app classification and perceived impact. In the present study, we only validated the main MARS components. Lastly, the goal of mHealth apps should be to improve health outcomes. As the present and previous studies show, few mHealth apps have been evaluated and assessed in medical studies. This means that most mHealth apps lack any evidence on health outcomes. In this sense, health outcome improvements through the use of mHealth apps need to be evaluated using standardized measures, such as randomized controlled trials. MARS can be used in conjunction with such studies to help determine the link between app quality and efficacy.

### Conclusion

A Japanese version of MARS was developed and shown to be as reliable and valid as the original MARS. The Japanese version of MARS can be used as a standard in evaluating the quality and credibility of mHealth apps. Further research is required for additional validation and for exploring the application of the scale in a range of research contexts.

## Appendix

Multimedia Appendix 1 Japanese version of the Mobile Application Rating Scale (MARS-Japanese).

## Abbreviations

ICC: intraclass correlation coefficient
MARS: Mobile App Rating Scale
mHealth: mobile health
