# Supplementary material for: Japanese Version of the Mobile App Rating Scale (MARS): Development and Validation
Source: JMIR Mhealth Uhealth. 2022 Apr 14;10(4):e33725. doi: 10.2196/33725 (PMC9052018; doi:10.2196/33725)
Supplement: Multimedia Appendix 1 [file mhealth_v10i4e33725_app1.pdf]

モバイルアプリ評価尺度（**MARS**）

この尺度の使用方法について

このスケールを用いて評価を行う方は

- 10分以上アプリを使用し、全ての機能を試してみてください。
- 使いやすさ、機能性、アプリの目的にかなっているかどうかを判断してください。
- アプリの設定、開発者の情報、外部リンク、セキュリティ機能などを確認してください。

スコアリングについて

「アプリの客観的品質」の評価はA～Dのスコアの平均を用います。「該当なし」と評価された質問は平均スコアの計算から除外してください。

「アプリの主観的品質」は、研究の目的に応じ個々の項目または全体の平均スコアとして使用することが出来ます。

「知覚的なインパクト」の項目を調整することで、健康行動に関するユーザーの知識・態度・意図に関するアプリの情報を得ることができます。

アプリの客観的品質（**A**～**D**）

A: エンゲージメント（愛用度） 平均スコア＝ ______

B: 機能性 平均スコア＝ ______

C: 見た目・デザイン性 平均スコア＝ ______

D: 情報 平均スコア＝ ______

アプリの客観的品質の平均スコア（= A + B + C + D / 4) : ______

アプリの主観的品質

E: アプリの主観的品質の平均スコア : ______

知覚的なインパクト

F: 知覚的なインパクトの平均スコア : ______

モバイルアプリ評価尺度（MARS）

アプリの分類

アプリの分類のセクションは、アプリに関する記述的および技術的な情報を収集するために使用してください。これらの情報を収拾するには、iTunes / Google Playに記載されているアプリの説明を確認してください。

アプリの名称:

このバージョンの星評価:

全バージョンの星評価 :

開発者:

このバージョンの評価者数:

全バージョンの評価者数:

バージョン:

最終更新日:

料金 - ベーシック版:

料金 - アップグレード版:

プラットフォーム: iPhone iPad Android その他

モバイルアプリ評価尺度（MARS）

<u>アプリの簡単な説明</u>

フォーカス：アプリが対象とするもの（該当するものすべて）

- ☐ 幸福感の向上
- ☐ 行動の変化
- ☐ ネガティブな感情の軽減
- ☐ 目標設定
- ☐ エンターテイメント
- ☐ マインドフルネス/瞑想/リラクゼーション
- ☐ アンガーマネージメント
- ☐ うつ病
- ☐ 不安・ストレス
- ☐ アルコール/薬物の使用
- ☐ 人間関係
- ☐ 身体の健康
- ☐ その他

____

理論的背景／戦略（該当するものすべて）

- ☐ 評価
- ☐ フィードバック
- ☐ 情報/教育
- ☐ モニタリング/トラッキング
- ☐ 目標設定
- ☐ アドバイス、ヒント、戦略、スキルトレーニング
- ☐ CBT-行動療法（ポジティブな出来事）
- ☐ CBT-認知療法（思考の挑戦）
- ☐ ACT - アクセプタンス・コミットメント・セラピー
- ☐ マインドフルネス/瞑想
- ☐ リラクゼーション
- ☐ 感謝の気持ち
- ☐ ストレングスベースドプラクティス（強みを生かす）
- ☐ その他

提携機関

不明　企業　政府　NGO　大学

年齢層（該当するものすべて）

- ☐ 子供（12歳未満）
- ☐ 青少年（13〜17歳）
- ☐ 若年層（18〜25歳）
- ☐ 成人
- ☐ 一般（全年齢層）

アプリの技術的側面（該当するものすべて）

- ☐ 連携可能（Facebook、Twitterなど）
- ☐ アプリのコミュニティがある
- ☐ パスワードによる保護が可能
- ☐ ログインが必要
- ☐ リマインダー機能
- ☐ ウェブアクセスが必要

モバイルアプリ評価尺度（MARS）

アプリの客観的品質評価

この評価スケールはアプリの品質を4つの次元で評価します。すべての項目は1から5までの5段階で評価されます。評価するアプリの構成要素の品質を最も正確に表す数字を丸で囲んでください。各回答に記載されている選択肢を使用してください。

セクション A：エンゲージメント（愛用度）

楽しい、興味が沸く、カスタマイズが可能、インタラクティブ・双方向性がある
（例：アラート、メッセージ、リマインダー、フィードバック、共有が可能）、
ターゲット層に合っている。

1. エンターテイメント性：そのアプリは使っていて楽しいか、面白いか。エンタテインメントを通じてエンゲージメントを高めるための戦略（ゲーミフィケーションなど）を採用しているか？

1 全く楽しくない
2 大部分が楽しくない
3 まあまあ楽しい。短時間（5分未満）であれば、楽しむことができる
4 楽しい。しばらくの間は楽しむことができる（合計5～10分程度）
5 大変楽しい。リピートしたくなるような面白さがある

2. 興味深さ：アプリを使っていて興味が湧くか？興味深い方法でコンテンツを提示することで、エンゲージメントを高める戦略を使っているか？

1 全然面白くない
2 大部分が面白くない
3 まあまあ面白い。短時間（5分未満）であれば興味を持つことができる
4 面白い。しばらく興味を持つことができる（合計5～10分）
5 非常に面白い。繰り返し使うことができる

3. カスタマイズ性：アプリの機能（サウンド、コンテンツ、通知など）に必要なすべての環境設定を提供/保持しているか？

1 カスタマイズができない。毎回設定を入力する必要がある
2 ほとんどカスタマイズができなので、アプリの機能に制限がある
3 基本的なカスタマイズが可能である
4 多様なカスタマイズが可能である
5 完璧にカスタマイズが可能である。ユーザーの特性や好みに合わせられる、設定を記憶させることができる

4. インタラクティビティ・双方向性：ユーザー入力が可能か、フィードバックがあるか、そのほか様々な機能（リマインダー、共有オプション、通知など）を含むか。（注：これらの機能が優れているためには、カスタマイズ可能かつ煩雑でないことが必要）

1　双方向の機能がない。またはユーザーの入力に反応しない
2　双方向の機能が不十分であり、アプリの機能が制限される
3　基本的な双方向性機能が備わっており、アプリは適切に作動する
4　さまざまな双方向の機能／フィードバック／ユーザー入力オプションがそなわっている
5　非常に高いレベルの双方向の機能／フィードバック／ユーザーの入力オプションがそなわっている

5. ターゲットとするグループ：アプリのコンテンツ（視覚情報、言語、デザイン）は、ターゲット層に適しているか？

1　完全に不適当、不明瞭あるいは混乱を招く
2　ほとんどが不適当、不明瞭あるいは混乱を招く
3　許容範囲だがターゲット向けにデザインされていない。時に不適当、不明瞭であり混乱を招く可能性がある
4　ターゲット向けにデザインされており、ほとんど問題ない
5　特にターゲット向けにデザインされており、全く問題ない

エンゲージメントの平均スコア＝ ________

セクション B：機能性

アプリの機能性、使いやすさ、ナビゲーション、ロジカルな構成、アプリの操作デザイン（タップ、スクロールなど）

6. パフォーマンス：アプリの特徴（機能）や構成要素（ボタンやメニュー）がどれだけ正確に／速く動くか？

1 アプリが壊れている。反応がない・不十分・不適切である（例：クラッシュ／バグ／機能が壊れている、など）
2 一部の機能は動作するが、タイムラグや重大な技術上の問題がある
3 アプリはおおむね動作するが、修正が必要な技術上の問題がある・動作が遅い時がある
4 大部分が正しく動作するが、マイナーな／無視できる程度の問題がある
5 完璧で素早く動作する。技術上のバグがない、「ローディング中」の表示がある

7. 使いやすさ：アプリの使い方を習得しやすいか、メニューのラベルやアイコン、説明がわかりやすいか。

1 使用説明がない／限られている。メニューのラベルやアイコンがわかりにくく複雑である
2 使用するのに多くの時間や手間がかかる
3 使用するのに多少の時間や手間がかかる
4 簡単に習得できる（またはわかりやすい使用説明がある）
5 すぐに使える、直感的でわかりやすい（使用説明がいらない）

8. ナビゲーション：画面間の移動が論理的／正確／適切／中断されないか、必要な画面へのリンクがすべてあるか。

1 アプリ内の他のセクションが論理的に切り離されているように見え、ランダムである/混乱する/ナビゲーションが難しい
2 習得に大変時間・労力がかかる
3 習得に少し時間・労力がかかる
4 習得しやすい・無視できる程度にリンクの無いものがある
5 完全に論理的・簡単・明快で、直感的な画面の流れである、またはショートカットがある

9. 操作デザイン：インタラクション（タップ／スワイプ／ピンチ／スクロール）は、すべての構成要素／スクリーンで一貫性があり、直感的に操作できるか？

1 全く一貫性がない、混乱をまねく
2 しばしば一貫性がない、混乱をまねく
3 許容範囲だが、時に一貫性がない、混乱をまねく
4 大部分に一貫性があり、直観的で、無視できる・些細な程度の問題しかない
5 完璧な一貫性と直感的な操作性である

機能性の平均スコア = ________

セクション C：見た目・デザイン性

グラフィックデザイン、全体的なビジュアル的アピール、配色、スタイルの一貫性

10. レイアウト：画面上のボタン、アイコン、メニュー、コンテンツの配置やサイズが適切か、必要に応じてズーム可能か。

1 デザインが非常に悪くわかりにくい。一部のオプションが選択・位置を確認・閲覧・読み取りしにくい。デバイスの表示が最適化されていない
2 デザインが悪く配置がバラバラでわかりにくい。一部のオプションが選択・位置を確認・閲覧・読み取りしにくい
3 許容範囲である。アイテムの選択・位置・閲覧・読み取りの問題はほとんどない
4 大部分がわかりやすい。アイテムの選択・位置の確認・閲覧・読み取りが可能である
5 デザインが非常に良い。シンプルではっきりしており、順序立てて論理的に表示されている。全てのデザインの要素に目的がある

11. グラフィックス：ボタン、アイコン、メニュー、コンテンツに使用されているグラフィックの品質／解像度はどの程度か？

1 グラフィックが素人レベルであり、見た目のデザインが非常に悪い。不釣り合いで、スタイル的に全く一貫性がない
2 低品質／低解像度のグラフィック、見た目のデザインが低品質である。不釣り合い、スタイル的に一貫性がない
3 まあまあの品質のグラフィックと見た目のデザインである（全体的に一貫したスタイルである）
4 高品質／高解像度のグラフィックと見た目のデザインである。ほとんどの配置、スタイルの均整が取れている
5 非常に高品質で解像度の高いグラフィックと見た目のデザインである。全体的に配置やスタイルの均整が取れている

12. 視覚的な魅力：アプリの見た目の良さは？

1 視覚的な魅力が全くない、見た目が不快感を催す、デザインが悪い、色が合っていない
2 視覚的な魅力が少ない - デザインが悪い、色使いが悪い、視覚的にぱっとしない
3 ある程度の視覚的魅力がある - 快適でも不快でもない、平均的
4 高い視覚的魅力がある - シームレスなグラフィック - 統一感のあるプロフェッショナルなデザイン
5 上記に加えて、非常に魅力的で、記憶に残り、目立ち、色使いがアプリの機能やメニューを引き立てている

見た目・デザイン性の平均スコア＝ ______

セクション D：情報

信頼できるソースからの高品質な情報（テキスト、フィードバック、測定値、参考資料など）であるか

*アプリの構成要素が該当しない場合は、「該当なし」を選択すること）

13. アプリの説明文の正確さ（アプリストア内）：アプリは説明されている内容を含んでいるか？

1　誤解を招く。説明されている構成要素や機能がアプリに含まれていない。または説明がない
2　不正確である。説明されている構成要素や機能がほとんど含まれていない
3　許容範囲。アプリには記載されている構成要素/機能の一部が含まれている
4　正確である。説明されている構成要素／機能のほとんどがアプリに含まれている
5　アプリの構成要素や機能が正確に説明されている

14. アプリの目標：アプリには、具体的で測定可能かつ達成可能な目標があるか（アプリストアの説明文やアプリ自体に明記されているもの）

該当なし　説明に目標が記載されていない、またはアプリの目標が研究目標とは無関係である（例：教育目的でゲームを使用する）
1　このアプリの利用により目標を達成する可能性はない
2　説明にはいくつか目標が記載されているが、アプリの利用により目標を達成する可能性はほとんどない
3　許容範囲である。目標が明確に設定されており、目標達成の可能性がある
4　目標が明確に設定されており、それらは計測・達成可能である
5　目標が明確に設定されており、達成できる可能性が高い

15. 情報の質：アプリのコンテンツは正しく、わかりやすく記述されているか、アプリの目標・テーマに関連したものであるか？

該当なし アプリ内に情報はない
1　まったく関連性がない／適切でない／一貫性がない／間違っている
2　ほとんど関連性がない／適切でない／一貫性がない／間違っている
3　まあまあ関連性がある／適切である／一貫性がある／正しく見える
4　大部分に関連性がある／適切である／一貫性がある／正確である
5　非常に関連性が高い／適切である／一貫性がある／正確である

16. 情報の量：アプリ内の情報は必要な内容が網羅されているか、包括的かつ簡潔に記述されているか。

該当なし アプリ内に情報はない
1 非常に少ない、あるいは非常に多い
2 かなり少ない、あるはかなり多い
3 許容範囲であるが、わかりにくい、あるいは簡潔でない
4 多くの情報があるが、バランスに欠ける。ときに不十分、あるいは不必要な情報がある、あるいはさらなる情報や情報元へのリンクがない
5 十分かつ簡潔で、さらなる情報や情報元へのリンクがある

17. ビジュアル的（視覚的）情報：コンセプトをチャート／グラフ／画像／ビデオなどを用いて明確・論理的・正確に説明しているか

該当なし アプリ内にビジュアル的な情報がない（例：音声やテキストのみの表示）
1 非常にわかりにくい／混乱を招く／間違っている、または必要なものが欠けている
2 ほとんどがわかりにくい／混乱を招く／間違っている
3 許容範囲だが、しばしばわかりにくい／混乱を招く／間違っている
4 ほとんどがわかりやすい／論理的／正確である、あるいは無視できる程度の問題のみ
5 非常にわかりやすい／論理的／正確である

18. 信頼性：アプリは信頼できる情報源に基づいているか（アプリストアの説明文やアプリ内に明記されているものについて）

1 情報元は特定されているが、情報の正当性/信頼性に疑問がある（例：個人情報の収集目的など）
2 正当な情報源のように見えるが、検証できない（例：趣味でのアプリ制作など）
3 小規模なNGO／機関（病院・公的機関など）／専門的な営利企業、助成団体による開発
4 大学などの組織が開発したもの、または3と同様だが規模が大きいものである
5 政府・研究機関の競争的資金（文科省科研費、厚労科研費など）を利用して開発されたものである

19. エビデンスに基づいているか：アプリの効果が検証されているか。エビデンス（公表されている科学的文献）で検証されるべき内容であるか

該当なし アプリは検証されていない
1. 検証により効果がないことが示唆されている
2. 無作為化比較試験（RCT）ではない研究にてアプリが検証され（例：受容性、使いやすさ、満足度評価）、部分的にポジティブな結果が得られている、あるいはエビデンスがほとんどない、あるいは矛盾するエビデンスは存在しない
3. RCTではない研究においてアプリが検証され（例：受容性、使い勝手、満足度評価）、ポジティブな結果が出ており、かつ他に矛盾した反証がない
4. アプリは1-2件のRCTで検証され、ポジティブな結果が得られている
5. アプリは3件以上の質の高いRCTで検証され、ポジティブな結果が出ている

情報の平均スコア= ______

*「該当なし」と評価された質問は、平均スコアの計算から除外してください。

アプリの主観的な品質

セクション E

20. このアプリを役に立ちそうな人に対して勧めたいと思いますか？

1. 全くそう思わない　このアプリは誰にも勧めない
2. 　このアプリを勧める人はほとんどいない
3. おそらくそう思う　このアプリを進めようと思う人はいる
4. 　このアプリを多くの人に勧めたい
5. 非常にそう思う　このアプリをみんなに勧めたい

21. このアプリがあなたに関係する内容であった場合、今後一年間（**12**ヶ月間）に何回利用すると思いますか？

1. 使用しない
2. 1-2
3. 3-10
4. 10-50
5. >50

22. このアプリが有料であったら利用しますか？

1. 払わない
2.
3.
4.
5. 払う

23. このアプリの総合的な星評価は？

1. ★　今まで使用したアプリの中で最も悪いものの一つ
2. ★★
3. ★★★　平均的
4. ★★★★
5. ★★★★★　今まで使用したアプリの中で最も良いものの一つ

モバイルアプリ評価尺度（MARS）

知覚的なインパクト

この項目を用いて、ユーザーの知識・態度・変化の意図だけでなく、実際の健康行動におけるアプリの知覚的なインパクトを評価することができます

セクション F

1. 自覚：このアプリは、[　対象となる健康行動を挿入　]に取り組むことへの自覚を高める可能性がある

   全くそう思わない　　　　　　　　　　　　　　　強くそう思う

   1　　2　　3　　4　　5

2. 知識：このアプリは、[　対象となる健康行動を挿入　]に関する知識／理解を深める可能性がある

   全くそう思わない　　　　　　　　　　　　　　　強くそう思う

   1　　2　　3　　4　　5

3. 態度：このアプリは、[　対象となる健康行動を挿入　]に対する態度を改善する可能性がある

   全くそう思わない　　　　　　　　　　　　　　　強くそう思う

   1　　2　　3　　4　　5

4. 変化の意図：このアプリは、[　対象となる健康行動を挿入　]に取り組む動機を高める可能性がある

   全くそう思わない　　　　　　　　　　　　　　　強くそう思う

   1　　2　　3　　4　　5

5. 支援の求め：このアプリを使用することで、[　対象となる健康行動を挿入　]のための支援を求めるようになると思われる（必要な場合）

   全くそう思わない　　　　　　　　　　　　　　　強くそう思う

   1　　2　　3　　4　　5

6. 行動の変化：このアプリを使用することで、[　対象となる健康行動を挿入　]を増加/減少させる可能性がある

   全くそう思わない　　　　　　　　　　　　　　　強くそう思う

   1　　2　　3　　4　　5
